# Is there any role for topical non-steroidal anti-inflammatory drugs in the treatment of mild to moderate musculoskeletal pain in a Lebanese community pharmacy?

**DOI:** 10.1186/s40064-016-2918-6

**Published:** 2016-08-02

**Authors:** Ahmad I. Dimassi, Mohamad K. Rahal, Mohamad M. Iskandarani, Etwal P. BouRaad

**Affiliations:** 1 Department of Pharmaceutical Sciences, School of Pharmacy, Lebanese International University, Mazraa, Beirut, 146404 Lebanon; 2 Department of Pharmacy Practices, School of Pharmacy, Lebanese International University, Mazraa, Beirut, 146404 Lebanon

**Keywords:** Topical, Systemic, Combination, NSAIDs, Mild to moderate musculoskeletal pain

## Abstract

**Background:**

Non-steroidal anti-inflammatory drugs (NSAIDs) are widely prescribed in the community pharmacy. Their systemic administration has been related to significant adverse effects. The aim of this study was to evaluate the efficacy of topical NSAIDs in managing mild to moderate musculoskeletal pain versus systemic administration or combination of both routes.

**Methods:**

This was a prospective observational study conducted in a large Lebanese community pharmacy over a period of 5 months in 302 patients. Participants were divided into three groups according to their route of administration of NSAIDs either topically, systemically or combination of both. During follow up period, degree of pain, efficacy of NSAID therapy, side effects, onset time and duration of pain relief reported by each patient were collected by the study investigators using specific formulated questionnaire. Degree of pain was assessed using the Numeric Rating Scale (NRS-11). The efficacy of NSAID therapy was defined as 50 % reduction or more in pain. The primary outcome was to assess the efficacy between groups. The secondary outcomes were onset time, duration of pain relief and side effects between the three groups.

**Results:**

A total of 149 patients were enrolled in this study. 78 patients administered topical NSAIDs, 40 administered systemic NSAIDs and 31 administered combination of both routes. Efficacy of NSAID therapy for all routes was reported in 132 participants (89 %) distributed as 64 in topical, 37 in systemic and 31 in combination. Bi-variate analysis showed no significant difference in efficacy between topical versus systemic (p = 0.99) and topical versus combination (p = 0.14). The mean onset of topical NSAIDs was significantly faster than systemic by 12.7 min (p < 0.05). The mean duration of pain relief of systemic NSAIDs was significantly longer than topical by 3 h (p < 0.05). Patients administered systemic NSAIDs (either alone or in combination) reported increase in blood pressure and gastric-upset 8 and 38 cases, respectively.

**Conclusion:**

There was no significant statistical difference between NSAIDs route’s of administration for the treatment of mild to moderate musculoskeletal pain in Lebanese community pharmacy patients. Topical NSAIDs were fast in onset and effective in reducing pain with no significant adverse effects.

## Background

 Non-Steroidal Anti-Inflammatory Drugs (NSAIDs) are among the most commonly used and effective medications to treat musculoskeletal conditions because of their anti-inflammatory and analgesic effects (Schnitzer et al. 2004; Mason et al. 2004; Zacher et al. 2001). Rasu et al. (2013) study showed that NSAIDs were the most preferred and prescribed drugs for chronic pain in the U.S. Although NSAIDs are effective and widely used for musculoskeletal pain, their adverse effects have become more evident (Vonkeman and Van de Laar 2010). Many studies on systemic use of NSAIDs have shown high incidence of serious gastrointestinal and cardiovascular adverse events (Lanza et al. 2009; Trelle et al. 2011; RodrÃguez et al. 2008; Johnson et al. 1994; Traversa et al. 1995). Topical NSAIDs were associated with lower side effects (Baraf et al. 2011); however their efficacy remains one of the most controversial issues in analgesia practice when compared to systemic NSAIDs (Klinge and Sawyer 2013). In the past 7 years, various updated guidelines such as National Institute for Health and Clinical Excellence (NICE) (Conaghan et al. 2008), European League Against Rheumatism (EULAR) (Smolen et al. 2010) and Osteoarthritis Research Society International (OARSI) (Zhang et al. 2008) have included recommendations for the use of topical NSAIDs, particularly in patients considered at gastrointestinal or cardiovascular high risk. A meta-analysis evaluated 34 studies from 7688 adults with chronic musculoskeletal pain found that topical NSAIDs provided adequate pain relief, equivalent to oral NSAIDs for hand and knee osteoarthritis (Derry et al. 2012). Due to lower bioavailability and plasma concentration compared to oral NSAID, topical administration provides local analgesia with lower incidence of systemic side effects (Stanos 2013; Miyatake et al. 2009). Nevertheless, the rate of local adverse effects is higher with topical NSAIDs, but they are reported to be minor self limited cutaneous reaction (Vaile and Davis 1998). According to our knowledge, there is a lack of sufficient evidence comparing oral to topical NSAIDs for the treatment of musculoskeletal pain conditions in Lebanon. The purpose of this study was to evaluate the efficacy of topical NSAIDs in the treatment of mild to moderate musculoskeletal pain versus systemic or combination of both routes in Lebanese community pharmacy patients.

## Methods

### Study design

This was a prospective observational study conducted from January 2014 till end of May 2014 in a large community pharmacy. All patients with a prescription of NSAID for the treatment of mild to moderate musculoskeletal pain were invited to participate in this study. Eligible patients were adults, had a prescription of NSAID to be administered either topically, systemically or in combination of both routes and complaining from acute (6 weeks or less of symptoms), sub-acute (7–12 weeks of symptoms) or chronic (more than 12 weeks of symptoms) (Goertz et al. 2012) mild (NRS 1–3) to moderate (NRS 4–6) musculoskeletal pain. Patients were excluded for the following reasons: severe musculoskeletal pain (NRS 7–10), cancer pain, headache, neuropathic pain, hypersensitivity to aspirin/NSAIDs or study participation refusal. The primary outcome of this study was to investigate the efficacy of NSAID therapy between various routes of administration, whereas the secondary outcomes were to assess the onset time of pain relief (in min), duration of pain relief (in h) and side effects between different NSAID’s routes of administration.

### Outcome measures

A structured questionnaire named “AD-23.16.14” was developed by the primary investigator and data were collected directly from the participants during face to face interview for maximum of 20 min. The questionnaire was piloted before starting the data collection in patients that shares the same characteristics as this study population. The severity of pain was assessed by the primary investigator using the Numeric Rating Scale (NRS-11) (MacCaffery and Beebe 1989). The efficacy measure of NSAID treatment was adopted from Moore et al. (1998) study, and defined as at least 50 % reduction in pain throughout NSAID therapy.

The onset time of pain relief was defined as the time required after administration of NSAID for a response to be observed (Mosby 2012). The duration of pain relief was defined as the length of time that NSAID is effective (McGraw-Hill 2002). The side effects of different NSAIDs routes were divided into local (dryness, itching and stinging) and systemic (increase in blood pressure and gastric upset) and they were assessed during follow-up dates by asking the patients to report any encountered side effects.

### Study procedure

A total of 302 patients were initially screened and the following data were recorded for each patient at baseline: patient’s age, gender, severity of pain, causes of pain, duration of pain (acute, sub-acute, chronic), site of pain, used analgesic in the last month, NSAID parameters (generic name, dose, frequency and dosage form), concomitant medications (anti-hypertensive, anti-diabetics and anti-lipidemics drugs), co-morbid diseases (hypertension, edema, ischemic heart disease, heart failure, peptic ulcer disease), history of previous gastrointestinal bleeding due to NSAIDs, and hypersensitivity to NSAIDs/aspirin. Patients who met the inclusion criteria were divided into three groups according to their NSAID’s route of administration including topical (gel, emulgel or difucrem), systemic (tablet, capsule, dispersible tablet, suppository, or injection) and combination of both topical and systemic dosage forms.

### Follow-up

All patients were followed up by the primary investigator on Day 3, D6, D10, D14, Week 4, W8 and W12 through phone calls or scheduled pharmacy visits. Follow up was terminated when the patient discontinued the NSAID treatment for a certain reason (effective therapy, failure of therapy, intolerable side effects or therapy withdrawal). During follow-up dates, the primary investigator counseled the patients about proper use of NSAIDs in terms of dose, frequency, and route of administration. Degree of pain, efficacy of NSAID therapy, onset time of pain relief (estimated in min), duration of pain relief (estimated in h) and side effects (local and systemic) were reported by each patient and collected by the study investigators using specific formulated questionnaire “AD-23.16.14”. All patients were asked about their therapy satisfaction, medication adherence, and add-on analgesics other than NSAID. Total duration of therapy was also recorded at the end of the treatment.

### Ethical considerations

Since our data collection methodology involves structured interview we obtained a signed informed consent form before starting the study. The consent form explained that all data will be stored in the School of Pharmacy at the Lebanese International University (LIU). Copies of the informed consent were kept as data research records in LIU School of Pharmacy. All data that is collected from our study has the potential to be shared publically. Consequently, to maximize benefits and minimize harm, investigators explained to all participants that any acquired information is to be treated with confidentiality and published results are to be in aggregate form with no reference to names.

### Statistical analysis

Descriptive statistics were used to describe patient characteristics (frequencies and percentages for categorical variables), and mean (±SD) for continuous variables.

Bi-variate analysis and cross-tabulation were used to identify the significance between different routes of administration and the efficacy of NSAID treatment. One way analysis of variance (ANOVA) test was performed to identify whether there is difference in onset time and duration of pain relief among the 3 groups. Statistical significance was defined as p value <0.05. Statistical analyses were performed using the Statistical Package for Social Science (SPSS version 20.0 IBM).

## Results

### Patient’s characteristics

**Fig. 1:**
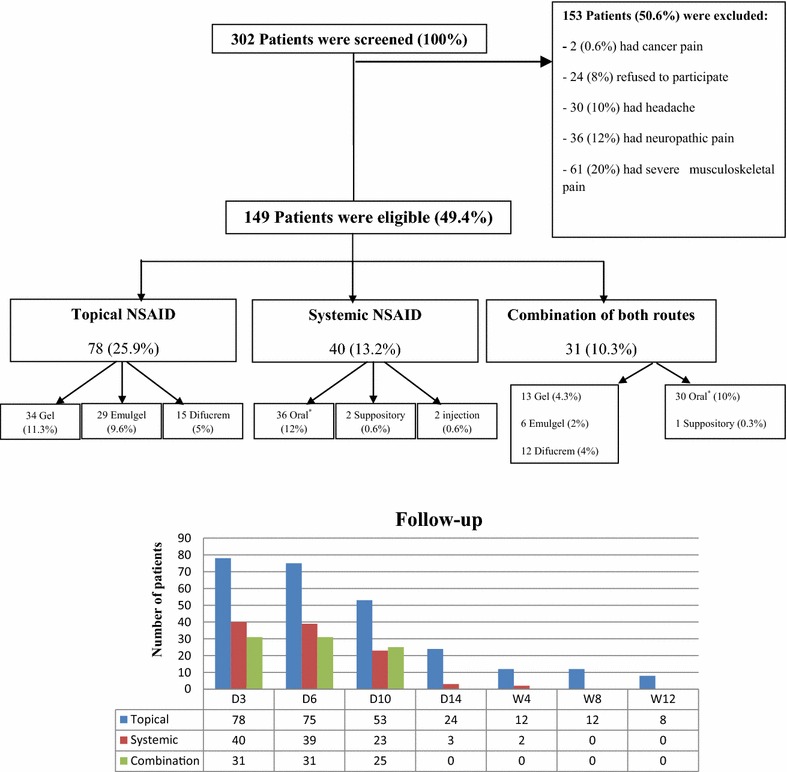
Patient’s enrollment and follow-up. *D* day, *W* week. *Oral dosage forms included: tablet, capsule and dispersible tablet. 302 patients were initially screened. A total of 149 patients fulfilled the inclusion criteria and were enrolled and followed up through phone calls or scheduled pharmacy visits. Enrolled patients were divided into 3 groups according to the route of administration of their NSAID therapy. Degree of pain, efficacy of NSAID therapy, onset time of pain relief, duration of pain relief and side effects were assessed during follow-up dates. At the beginning all patients used NSAID for 3 days, afterward the number of patients decreased due to withdrawal of NSAID therapy for many reasons such as efficacy, lack of efficacy or adverse effects. Topical NSAID were used up to 12 weeks, systemic NSAID up to 4 weeks and combination of both routes up to 10 days

A total of 302 patients were initially screened, 153 patients (50.6 %) were excluded for the following reasons: severe musculoskeletal pain (20 %), neuropathic pain (12 %), headache (10 %), study participation refusal (8 %) and cancer pain (0.6 %). The remaining 149 (49.4 %) patients were enrolled and followed up in this study (Fig. 1).

Patient’s characteristics are shown in Table 1. Enrolled patients had a mean age of 40.7 ± 15.96 (mean years ± SD). From the total number of enrolled patients (n = 149), 72 (48 %) participants were males and 77 (52 %) were females. Majority of the patients (78 %) had moderate musculoskeletal pain.

**Table 1:** Patient’s characteristics

Characteristic	Value
Age (years)	
Mean ± SD (range)—years	40.7 ± 15.96 (12–92)
Gender—no. (%)	
Male	72 (48)
Female	77 (52)
Severity of pain—no. (%)	
Mild	33 (22)
Moderate	116 (78)
Duration of pain—no. (%)	
Acute	100 (67)
Sub-acute	37 (25)
Chronic	12 (8)
Co-morbid diseases—no. (%)	
Hypertension	45 (30)
CAD	23 (15)
Diabetes	12 (8)
PUD	12 (8)
Concomitant medications with NSAIDs—no. (%)	
Anti-hypertensives	49 (33)
PPIs	34 (23)
Anti-lipidemics	24 (16)
Anti-diabetics	13 (9)
Concomitant analgesics with NSAIDs—no. (%)	
Muscle relaxant	20 (13.5)
Topical group	7 (35)
Systemic group	11(55)
Combination group	2 (10)
Acetaminophen	12 (12.5)
Topical group	9 (75)
Systemic group	1 (9)
Combination group	2 (16)
Vitamin B complex	7 (5)
Topical group	0 (0)
Systemic group	2 (29)
Combination group	5 (71)
Sites of pain—no. (%)	
Back	42 (28)
Shoulder	27 (18)
Multiple sites*	21 (14)
Legs	15 (10)
Neck	13 (9)
Ankle	12 (8)
Hands	6 (4)
Knees	6 (4)
Fingers	4 (3)
Elbow	3 (2)
Used NSAIDs agents—no. (%)	
Diclofenac	76 (51)
Ketoprofen	45 (30)
Aceclofenac	37 (25)
Naproxen	18 (12)
Piroxicam	4 (2)
Etoricoxib	1 (0.5)

Mild pain category included all patients with NRS 1–3; Moderate pain category included all patients with NRS 4–6

Acute pain: <6 weeks of symptoms, sub-acute pain: 7–12 weeks of symptoms, chronic pain: >12 weeks of symptoms

Anti-hypertensives included ACE inhibitors, CCBs, beta-blockers; PPIs included: omperazole, lanzoprazole and esomeprazole

Anti-lipidemics included statins and fibrates; anti-diabetics included sulfonylureas, metformin and insulin

Muscle relaxant included orphenadrine, chlorzoxazone and magnesium supplements

*no.* number, *%* percentage, *SD* standard deviation, *CAD* coronary artery disease, *PUD* peptic ulcer disease, *PPI* proton pump inhibitor

* Multiple sites variable is defined as involvement of 2 or more sites of pain

Acute pain was the predominant duration of pain (67 %) followed by sub-acute (25 %) and chronic (8 %). Co-morbid diseases were reported by the participants at baseline such as hypertension (30 %), coronary artery disease (15 %), diabetes (8 %) and peptic ulcer disease (8 %). Consequently, anti-hypertensives (33 %), proton pump inhibitors (23 %), anti-lipidemics (16 %) and anti-diabetics (9 %) were used by the participants.

Co-analgesics were used in addition to NSAID therapy by a total of 39 patients, of which 20 used muscle relaxants, 12 used acetaminophen and 7 used vitamin B complex supplements.

Back pain was the most common site of musculoskeletal pain as reported by 42 patients (28 %), followed by shoulder (18 %), leg (10 %), neck (9 %), ankle (8 %), hand (4 %), knee (4 %), finger (3 %) and elbow pain (2 %).

A total of 76 patients (51 %) had a prescription of diclofenac, followed by ketoprofen 45 (30 %), aceclofenac 37 (25 %), naproxen 18 (12 %), piroxicam 4 (2 %) and etoricoxib 1 (0.5 %).

### Primary outcome

#### Efficacy of NSAID therapy among the three groups

**Fig. 2:**
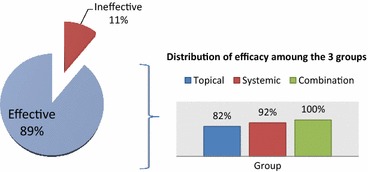
Overall efficacy of NSAID therapy and its distribution among the 3 groups. Overall efficacy of NSAID therapy is considered in all routes of administration (149 patients) when patients reported at least 50 % reduction of pain. Bi-variate analysis and cross-tabulation between different routes of administration and the efficacy of NSAID treatment were done to identify if there is a significant difference of efficacy when distributed among the 3 groups. No significant difference in efficacy between topical versus systemic (p value = 0.99) and topical versus combination of NSAID therapy (p value = 0.14)

The overall efficacy rate of NSAID therapy in mild to moderate musculoskeletal pain was reported in 132 patient out of 149 (89 %), distributed as 64 out of 78 patients (82 %) in topical, 37 out of 40 patients (92 %) in systemic, and 31 out of 31 patients (100 %) in combination group as shown in (Fig. 2). Bi-variate analysis and cross-tabulation between different routes of administration and the efficacy of NSAID treatment showed no significant difference in efficacy between topical versus systemic NSAID therapy (p value = 0.99) and topical versus NSAID combination therapy (p value = 0.14).

### Secondary outcomes

#### Onset time of pain relief

The mean onset time of pain relief was 17.3 min (±7.19 SD) in the topical, 30 min (±10.3 SD) in the systemic and 14.5 min (±3.87 SD) in the combination NSAID therapy group. Topical NSAIDs were significantly faster than systemic NSAIDs by 12.7 min with p value of the mean difference <0.05, but slower than in combination group by 2.8 min with p value = 0.28. The mean onset of pain relief in combination therapy was significantly faster than systemic by 15.5 min with p value of the mean difference <0.05 (Table 2).

**Table 2:** Mean onset time of pain relief (estimated in minutes) among the 3 groups

Mean onset in each group (min) “i”	Mean onset in each group (min) “j”	Mean onset difference among groups (min): “i − j”	p value	95 % CI	
Topical (17.3)	Systemic (30)	−12.7	0.001*	−16.3631	−9.1241
	Combination (14.5)	2.8	0.28	−1.1952	6.7080
Systemic (30)	Topical (17.3)	12.7	0.001*	9.1241	16.3631
	Combination (14.5)	15.5	0.001*	11.0464	19.9536
Combination (14.5)	Topical (17.3)	−2.8	0.28	−6.7080	1.1952
	Systemic (30)	−15.5	0.001*	−19.9536	−11.0464

Onset time of pain relief is defined as the time required after administration of NSAID for a response to be observed. Patients required a mean of 17.3 min to feel pain relief in topical NSAID group, 30 min in systemic NSAID group and 14.5 min in combination group

Mean onset difference among groups was calculated by subtracting mean onset of each 2 groups (i−j). One way analysis of variance (ANOVA) test was performed to assess if there is a significant difference in the mean onset time of pain relief among groups. Topical NSAIDSs were significantly faster than systemic by 12.7 min (p value <0.05, 95 % CI). Combination therapy was significantly faster than systemic by 15.5 min (p value <0.05, 95 % CI). Topical NSAIDs were insignificantly slower than combination group by 2.8 min (p value = 0.28, 95 % CI)

* The mean onset difference among groups is significant at p value <0.05 and 95 % confidence interval

#### Duration of pain relief

The mean duration of pain relief was 10 h (SD ± 3.74) in the systemic, 7 h (SD ± 3.46) in the topical and 11 h (±2.05 SD) in the combination NSAID therapy group. The mean duration of pain relief in systemic NSAIDs group was significantly longer than topical by 3 h with p value of the mean difference <0.05, but shorter than combination therapy by 1 h with p value of the mean difference = 0.85. The mean duration of pain relief in combination group was significantly longer than topical group by 4 h with p value of the mean difference <0.05 (Table 3).

**Table 3:** Mean duration of pain relief (estimated in hours) among the 3 groups

Mean duration of pain relief in each group “i”	Mean duration of pain relief in each group “j”	Mean duration of pain relief difference among groups (hours) “i − j”	p value	95 % CI	
Topical (7)	Systemic (10)	−3	0.001*	−4.6341	−1.5172
	Combination (11)	−4	0.001*	−5.6303	−2.2274
Systemic (10)	Topical (7)	3	0.001*	1.5172	4.6341
	Combination (11)	−1	0.85	−2.7708	1.0644
Combination (11)	Topical (7)	4	0.001*	2.2274	5.6303
	Systemic (10)	1	0.85	−1.0644	2.7708

Duration of pain relief is defined as the length of time that NSAID is still effective. Patients reported a mean of 7 h of pain relief in topical NSAID group, 10 h in systemic NSAID group and 11 h in combination NSAID group

Mean duration of pain relief difference among groups was calculated by subtracting mean duration of pain relief of each 2 groups (i − j). One way analysis of variance (ANOVA) test was performed to assess if there is a significant difference in the mean duration of pain relief among groups. Duration of pain relief in systemic NSAID group was significantly longer than topical NSAID group by 3 h (p value <0.05, 95 % CI). Duration of pain relief in combination group was significantly longer than topical group by 4 h (p value <0.05, 95 % CI) and insignificantly longer than systemic group by 1 h (p value = 0.85, 95 % CI)

* The mean duration of pain relief difference among groups is significant at p value <0.05 and 95 % confidence interval

#### Side effects of NSAID therapy among the 3 groups

During the follow-up period, patients were asked by the primary investigator to report any encountered side effect. Adverse effects were divided into local and systemic. Patients who administered systemic NSAIDs (either alone or in combination) reported increase in blood pressure and gastric-upset 8 and 38 cases respectively. Whereas patients who administered topical NSAIDs reported mild transient local dermatological side effects, such as dryness, itching and stinging 2, 3 and 2 cases respectively (Table 4).

**Table 4:** Reported systemic and local side effects in the 3 groups

*Systemic SE*	
Increase in blood pressure—no.	
Topical	0
Systemic	7
Combination	1
Gastric upset—no.	
Topical	0
Systemic	23
Combination	15
*Local SE*	
Dryness—no.	
Topical	2
Systemic	0
Combination	0
Itching—no.	
Topical	3
Systemic	0
Combination	0
Stinging—no.	
Topical	2
Systemic	0
Combination	0

Local and systemic side effects were assessed during follow-up dates. Gastric upset was characterized by epigastric pain, heartburn, stomach discomfort, bloating, nausea and vomiting

*no* Number of enrolled patients, *SE* side effects

## Discussion

In this study, NSAIDs showed to be effective in the treatment of mild to moderate musculoskeletal pain by 89 %. Roelofs et al. (2008) study has also shown that NSAIDs were the mainstay treatment for acute and chronic musculoskeletal pain.

The primary outcome of this study was to assess the efficacy of various NSAIDs routes in musculoskeletal pain. When efficacy of NSAID therapy was compared among the 3 groups, all patients (100 %) in the combination group had efficacy of NSAID therapy, followed by 92 % in the systemic group and 82 % in the topical group. Bi-variate analysis and cross-tabulation showed no significant difference between the 3 groups in term of efficacy with a p values of 0.99 between topical versus systemic group and 0.14 between topical versus combination group. Three clinical trials, with 764 patients, compared topical NSAIDs with oral NSAIDs (diclofenac 100 mg daily in one trial and ibuprofen 1200 mg daily in two). The overall efficacy rate was similar for topical NSAIDs (37 %) and oral NSAIDs (37 %) with no statistically significant difference (Bookman et al. 2004). These results were similar to that in our study. However, Curatolo and Bogduk (2001) used a pragmatic review of data provided by available systematic reviews and seminal controlled studies pertaining to the treatment of musculoskeletal pain concluded that NSAIDs have limited effectiveness in the treatment of musculoskeletal pain.

In this study, patients who administered the NSAID therapy for more than 4 weeks were in the topical group and had chronic musculoskeletal pain; during follow-up dates, these participants reported that they were satisfied with the results of the topical NSAID till the end of the study. Similarly, Underwood et al. (2008) compared topical versus oral NSAIDs in chronic knee pain and concluded that topical NSAIDs are as effective as oral in knee pain for 12 months; this reflects that topical NSAIDs could retain their efficacy when used chronically.

According to our knowledge, there are no other studies that investigated the onset time of pain relief and duration of pain relief of NSAID therapy in community pharmacy patients. This study showed that topical NSAIDs were significantly faster in onset (mean = 17.3 min) compared to systemic therapy (mean = 30 min), this could be due to the direct and local effects of topical dosage form on the affected area. Duration of pain relief in systemic NSAIDs group (mean = 10 h) was significantly longer than topical NSAIDs group (mean = 7 h) this could be explained by the accumulation of NSAID in the synovial fluids and soft tissues, that would increase the time to clear the drug from these sites. What is important about combination of both NSAIDs routes that it combined the fast onset of topical and the long duration of pain relief of systemic NSAIDs and ended up with a remedy of rapid onset (mean = 15 min) and extended duration of action (mean = 11 h).

The addition of other analgesics like acetaminophen, muscle relaxants and vitamin B complex were reported. These co-analgesics interfered with the efficacy of the NSAID treatment and could be considered as confounding variables. It was noted that vitamin B complex and muscle relaxants were frequently administered in the systemic group, whereas acetaminophen was mainly taken in the topical NSAIDs group.

In our study, systemic side effects such as increased blood pressure and gastrointestinal upset were reported in 8 and 38 participants respectively, these participants were taking NSAIDs systemically either alone or in combination. Surprisingly, the rate of these systemic side effects was lower in the combination group compared to systemic group; this could be explained by the lower doses and frequencies of the used systemic NSAIDs when combined with the topical NSAIDs. A study done by Miyatake et al. (2009) showed that plasma levels resulting from topical and oral applications of diclofenac were comparable; however, after topical application the concentration levels were higher in the muscle and lower in the synovial tissues of osteoarthritis patients compared with orally treated patients. Conversely, a comprehensive review article done by Klinge and Sawyer (2013) in which topical NSAIDs and oral NSAIDs were compared in the treatment of both acute and chronic musculoskeletal injury, the authors concluded that caution should be exercised with the use of both topical and oral NSAIDs, particularly in patients with previous adverse reactions to NSAIDs.

We found that most of the patients had applied topical NSAIDs for a total of 10 days, those patients reported high rates of efficacy and mild self limited local adverse events described as dryness, itching and stinging. Similarly, Baer et al. (2005) and Bookman et al. (2004) reported dry skin as the most common local adverse event of the topical NSAID therapy. Additionally, several studies have compared topical with oral NSAIDs and concluded that topical NSAIDs were effective and safe in treating acute painful conditions for one week, and patients preferred to use topical NSAIDs as gastrointestinal adverse events limited the use of oral NSAIDs (Dickson 1991; Rother et al. 2007; Tugwell et al. 2004; Mason et al. 2004; Derry et al. 2015). Accordingly, topical NSAIDs could be a reasonable option to treat mild to moderate musculoskeletal pain as it provides good efficacy with no systemic side effects, especially in patients with hypertension and gastrointestinal diseases.

### Strengths and limitations

This is the first descriptive study that was specifically designed to assess the efficacy, onset time of pain relief and duration of pain relief of topical, systemic or combination of both routes of NSAIDs in Lebanese community pharmacy patients.

One of the major limitations was the patient’s assessment of pain, since it is a personal feeling and it could vary between subjects. Another limitation of the study was the use of different NSAIDs agents for a short period of time; consequently many long term outcomes couldn’t be analyzed, this would explain the findings of few side effects of the NSAID therapy especially the systemic ones in the combination group. Baseline characteristics and classification of the participants into three groups further decreased the sample size and affected the results. Additionally, the study was conducted at one community pharmacy in Lebanon which also justifies the small number of enrolled patients in this study and the inability of results generalization. It would therefore be recommended to conduct additional studies including large sample size of patients taking NSAIDs for long period of time for the treatment of musculoskeletal pain in all areas of Lebanon.

## Conclusion

NSAIDs are the cornerstone of musculoskeletal pain treatment, but their systemic use may be limited in certain patients due to their potential adverse effects. According to our study, there was no significant statistical difference of efficacy between topical, systemic or combination of both routes of NSAIDs in the treatment of mild to moderate musculoskeletal pain in Lebanese community pharmacy patients. Topical NSAIDs were fast in onset and effective in reducing pain with no significant systemic adverse effects. Combination of topical and systemic NSAIDs routes showed faster onset and long duration of pain relief, but it was associated with systemic adverse effects. What remains unclear is the safety of NSAIDs combination of both routes. Additionally it was uncertain if topical NSAIDs remain safe and retain their efficacy on the long term. Thereby, additional large-scale, long-term studies in patients with musculoskeletal pain are needed to identify the role of topical NSAIDs when combined with oral NSAIDs.
